# Efficacy of Chemiluminescence Immunoassays on VCA-IgA and EBNA1-IgA Antibodies of Epstein-Barr Virus in Diagnosing Nasopharyngeal Carcinoma

**DOI:** 10.7150/jca.47260

**Published:** 2020-10-18

**Authors:** Xia Yu, Fugui Li, Weimin Cheng, Biaohua Wu, Huiyun Fang, Fuzhen Xia, Yijun Gong, Wenjing Yu, Pu Liao, Youde Cao, Fenghua Yang, Hong Zhu, Jiang Li, Yajun Huang, Liying Gan, Lei Zhang, Yonggang Lou, Mingfang Ji

**Affiliations:** 1 Cancer Research Institute of Zhongshan City, Zhongshan City People's Hospital, Zhongshan, Guangdong, China; 2 Guangdong Provincial Engineering Technology Research Center for Autoimmune Laboratory Diagnostic Products, Shenzhen, Guangdong, China; 3 Department of Clinical Laboratory, First Hospital of Jilin University, Changchun, Jilin, China; 4 Department of Clinical Laboratory, Chongqing General Hospital, Chongqing, China; 5 Department of Clinical Laboratory, Hunan Provincial People's Hospital (The First Affiliated Hospital of Hunan Normal University), Changsha, Hunan, China; 6 Department of Clinical Laboratory, Fushun Central Hospital, Fushun, Liaoning, China; 7 Department of Clinical Laboratory, The First Affiliated Hospital of Dalian Medical University, Dalian, Liaoning, China; 8 Department of Clinical Laboratory, Shenzhen Sixth People's Hospital (Nanshan Hospital), Huazhong University of Science and Technology Union Shenzhen Hospital, Shenzhen, Guangdong, China; 9 Department of Clinical Laboratory, The Second Affiliated Hospital of Fujian Medical University (Donghai Hospital District), Quanzhou, Fujian, China; 10 Department of Clinical Laboratory, Guangxi International Zhuang Medicine Hospital, Nanning, Guangxi, China; 11 Department of Clinical Laboratory, Capital Medical University Daxing Teaching Hospital, Beijing, China; 12 Department of Clinical Laboratory, Dongyang People's Hospital, Dongyang, Zhejiang, China

**Keywords:** nasopharyngeal carcinoma, VCA-IgA, EBNA1-IgA, CLIA, ELISA

## Abstract

**Background:** IgA antibodies against Epstein-Barr virus (EBV) capsid antigen (VCA) and nuclear antigen 1 (EBNA1) have been proposed to facilitate the diagnosis and early detection of nasopharyngeal carcinoma (NPC) in high-incidence regions. However, while new methodologies and new platforms for the detection of VCA-IgA and EBNA1-IgA have become available, proper interassay simultaneous comparisons have not been carried out. The study was to compare the performance of the chemiluminescent immunoassays (CLIA) and enzyme-linked immunosorbent assay (ELISA) for VCA-IgA and EBNA1-IgA antibodies, and to evaluate the levels of EBV antibodies in healthy population from different areas of China.

**Methods:** CLIA and ELISA for VCA-IgA and EBNA1-IgA were performed in NPC and healthy populations from high-incidence areas of NPC in South China (*N*=555), medium-incidence areas of NPC in Central China (*N*=318) and low-incidence areas of NPC in North China (*N*=379), and the results were compared and analyzed.

**Results:** (1) The highest sensitivity in total, early and advanced NPC were 91.5% (CLIA for VCA-IgA), 86.4% (CLIA and ELISA-2 for EBNA1-IgA) and 93.6% (CLIA for VCA-IgA). However, the specificity of EBV-IgA measured by CLIA was relatively lower than ELISA. The top three seromarkers with the largest AUC was CLIA for VCA-IgA (AUC: 0.929, 95% CI: 0.905-0.953), ELISA-2 for EBNA1-IgA (AUC: 0.922, 95% CI: 0.896-0.947) and CLIA for EBNA1-IgA (AUC:0.919, 95% CI: 0.893-0.945), respectively. The positive and negative coincidence rates of the two EBNA1-IgA kits were 69.5% and 91.9%, respectively. However, the coincidence rates of VCA-IgA were relatively low. CLIA kits had good repeatability between different laboratories. (2) The positive rates of EBV-IgA antibodies were relatively high in high-incidence areas of NPC (*P <* 0.017), while there was no significant difference in the antibody positive rates between medium-incidence areas and low-incidence areas of NPC (*P >* 0.05).

**Conclusions:** The performance of EBV-IgA antibodies measured by CLIA has good repeatability, higher sensitivity and similar specificity. The higher EBV-IgA positive rate in healthy subjects by CLIA raises concern about its suitability for NPC-risk screening and requires further analysis.

## Introduction

Nasopharyngeal carcinoma (NPC) is a common malignant tumor in the Southern China, which is closely related to Epstein-Barr virus (EBV) infection 1, 2. Individuals with elevated levels of antibody responses against EBV antigens (particularly IgA responses) are at increased risk for development of NPC 3-6. At present, enzyme-linked immunosorbent assay (ELISA) combined detection of IgA antibodies against EBV capsid antigen (VCA-IgA) and nuclear antigen 1 (EBNA1-IgA) has been proposed for general population screening to triage individuals for further clinical evaluation 7-10. ELISA-based assays are easier to be standardized, with greater false tolerance to interference, and are also more labor-saving when analyzing a large number of specimens. Chemiluminescent immunoassay (CLIA) has advantages similar to ELISA 11, but few literatures of CLIA testing EBV-IgA has been reported yet. In addition, while new methodologies and new platforms (ELISA vs.CLIA) for the detection of VCA-IgA have become available, proper interassay simultaneous comparisons have not been carried out. Here, CLIA was employed to detect VCA-IgA and EBNA1-IgA for the first time in this study, and compared with several ELISA kits widely marketed for diagnostic performance analysis of NPC. Meanwhile, the difference of EBV antibody positive rate among healthy population in high-incidence areas, medium-incidence areas and low-incidence areas of NPC in China was compared in order to provide relevant scientific basis for clinical application of kit.

## Materials and Methods

### Subjects

The subjects were divided into NPC group and healthy group. All subjects collected 2-4 mL fasting venous blood. After centrifugation, the serum samples were stored at 4°C for use within one month, or stored at -80°C for longer periods. The inclusion criteria of NPC group included the following: being aged 20-69 years, pathological examination confirmed undifferentiated non-keratinized carcinoma and untreated. NPC group were continuously collected from 201 patients with NPC hospitalized in Zhongshan City People's Hospital from March 2019 to December 2019. Among them, 153 were males and 48 were females, and the ratio of males to females was 3.19:1. The mean age was 49.18 ± 12.23 years with the median age 48 years. NPC staging was based on the 8^th^ edition of UICC staging, including 44 patients of early (I + II) NPC and 157 advanced (III + IV) NPC.

A total of 1,051 healthy subjects, who were 20-69 years old, were randomly selected among healthy people who participated in physical examinations at hospital from March 2019 to December 2019, and their serum samples came from 11 hospitals in different regions of China. We excluded samples from immunocompromised patients (e.g.,those with cancer, organ transplant recipients, or those with other infectious diseases). The classification criteria for different incidence areas of NPC is based on the *2018 China Cancer Registry Annual Report*^12^. The population came from the following three areas: (1) High-incidence areas of NPC: Zhongshan and Shenzhen in Guangdong, Nanning in Guangxi, with 354 people, including 171 males and 183 females, with male: female = 0.93: 1. The mean age was 43.52 ± 14.19 years with the median age 43 years. (2) Medium-incidence areas of NPC were Changsha in Hunan, Quanzhou in Fujian, Dongyang in Zhejiang, and Chongqing, with 318 people in total, including 165 males and 153 females, with male : female =1.08 : 1. The mean age was 45.10 ± 13.08 years, with the median age 46 years. (3) Low-incidence areas of NPC included Beijing, Changchun in Jilin, Dalian and Fushun in Liaoning, with 379 people in total, including 190 males and 189 females, with male: female = 1.01:1. The mean age was 44.70 ± 14.14 years with the median age was 46 years. There was no significant difference in sex and age among the three groups of healthy population (*χ^2^* =0. 861, *P=*0. 650, *P>*0.05; *F*=2. 338, *P=*0. 311, *P>*0.05).

### Reagents and Methods

1.2.1 CLIA reagent VCA-IgA and EBNA1-IgA kits are manufactured by Shenzhen YHLO Biotech Co., Ltd. The iFlash 3000 chemiluminescence immunoanalyzer and matching reagent (Acridine Ester Direct Chemiluminescence) were employed. Following the manufacturer's instruments, reagents and standard operating procedure (SOP), the two test results are expressed by COI value with COI ≥ 1.1 stands for reacted (positive).

1.2.2 ELISA reagent for VCA-IgA kit is manufactured by Euroimmun Medizinische Labordiagnostika AG (represented as ELISA-1). ELISA reagent VCA-IgA and EBNA1-IgA kits are manufactured by Zhongshan Bioengineering Co., Ltd (represented as ELISA-2). The levels of these seromarkers were assessed by photometric measurement, according to manufacturer's instructions, and standardized by calculating the ratio of the optical density (OD) of the sample over that of the reference control (rOD). If the specific rOD was greater than 1, the sample was regarded as positive.

1.2.3 VCA-IgA of ELISA-1 was purified VCA gp125 from P3HR1 cells, while ELISA-2 and CLIA kits used recombinant VCA protein components (p18 and p23). EBNA1-IgA of ELISA-2 and CLIA were produced with purified recombinant peptides specified by EBV BKRF1 (72kDa). All the serological tests were conducted by the same technicians (sample information was blinded) .Serial dilutions of quality control sera and negative control were applied to each assay for the evaluationg of intra-variability.

### Evaluation of Detecting Performance

Sensitivity= number of people with positive antibody tests in NPC/total number of NPC; Specific= number of people with negative antibody tests in healthy population/total number of healthy population; Positive coincidence rate= a / (a + b + c), negative coincidence rate = d / (b + c + d). Where a represents the number of positive detection results of both detection systems; b indicates the number of positive cases detected by the X detection system but negative by the Y detection system; c indicates the number of negative cases detected by the X detection system but positive by the Y detection system; d represents the number of negative tests by both detection systems. Parallel detection: If one of the two indicators is positive, it will be determined as positive, and if all indicators are negative, it will be determined as negative. Laboratories in different hospitals were randomly selected to use CLIA kits to detect EBNA1-IgA and VCA-IgA, and to evaluate the consistency of tests results between different laboratories.

### Statistical Analysis

SPSS19.0 software was used for analysis, and the counting data were expressed as percentages, and chi-square test was used for comparison of data between groups. The variables were tested for normality, and the skewness distribution data were tested under rank sum of independent samples. The area under curve (AUC) was calculated by the thereceiver operating characteristic curve (ROC) to evaluate the diagnostic efficacy of EBV in NPC. The level of significant test was α = 0.05, with *P<*0.05 deemed significant.

## Results

### Analysis of Positive Rate of EBV Antibody Detected by CLIA and ELISA in Healthy Population

CLIA and ELISA were used to detect VCA-IgA and EBNA1-IgA antibodies in healthy population in high-incidence areas, medium-incidence areas and low-incidence areas of NPC, respectively, and the positive rates of healthy population were calculated respectively, detailed in Table 1. The positive rates of CLIA, ELISA-1 and ELISA-2 for VCA-IgA varied between three kits, and the positive rates of total population were 5.6%, 2.5% and 7.3%, respectively. However, the positive rates of CLIA and ELISA-2 reagents for EBNA1-IgA were similar with 5.6% and 5.2%, respectively. In healthy population, the positive rate of EBV-IgA measured by CLIA was higher than ELISA.

EBNA1-IgA by CLIA and ELISA-2, and VCA-IgA by ELISA-1 revealed that there were significant differences in the three positive rates of EBV antibody in three different areas (*P*< 0.05). The results showed that the EBNA1-IgA positive rates of CLIA and ELISA-2 were 8.5% and 8.8%, respectively, and the VCA-IgA positive rate by ELISA-1 was 4.8% in healthy people in high-incidence areas, and the antibody positive rate was higher in healthy population in high-incidence areas (*P <* 0.017), while there were no significant differences in the antibody positive rates between medium-incidence areas and low-incidence areas of NPC (*P >* 0.05).

### Comparison of Efficacy of CLIA and ELISA in Detecting EBV Antibody in Diagnosis of NPC

CLIA and ELISA were used to detect VCA-IgA and EBNA1-IgA antibodies in NPC and healthy population, and the sensitivity and specificity of various EBV antibodies in diagnosing NPC were calculated respectively. In total NPC and advanced NPC, CLIA detected the highest VCA-IgA sensitivity with 91.5% and 93.6% respectively. However, in early NPC, the EBNA1-IgA sensitivity of CLIA and ELISA-2 reached 86.4%. Among five kits, the highest specificity was ELISA-1, as shown in Table 2.

**Fig 1:**
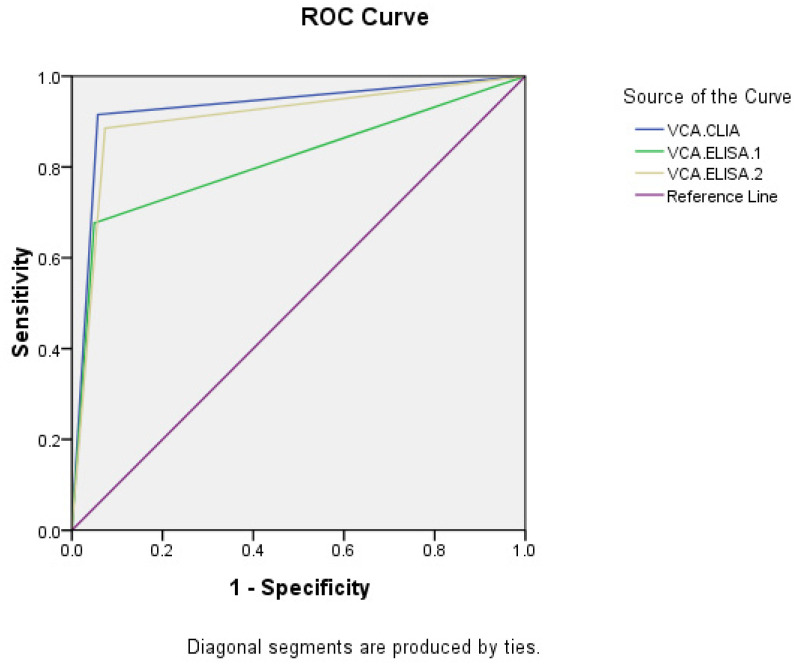
ROC of VCA-IgA in diagnosis of NPC

**Fig 2:**
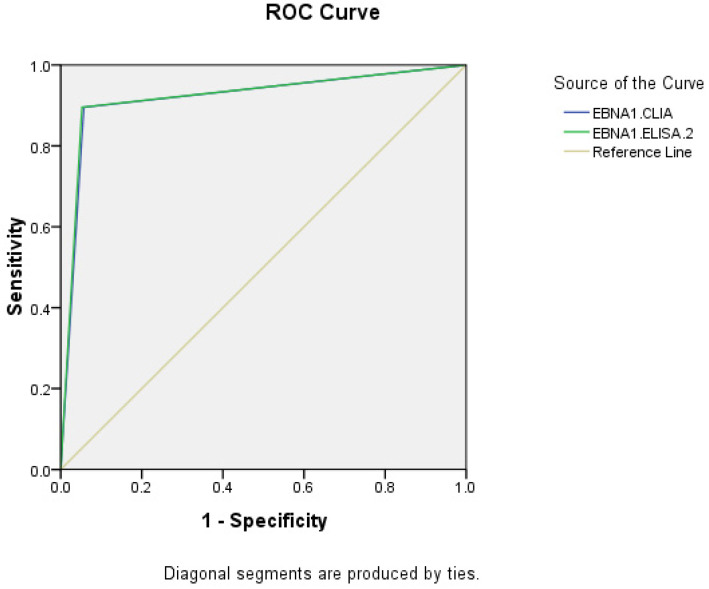
ROC of EBNA1-IgA in diagnosis of NPC

Taking NPC and healthy people as research objects, ROC curves were made (Figures 1, 2). The AUC of VCA-IgA in diagnosis of NPC by CLIA, ELISA-1 and ELISA-2 were 0.929 (95% CI: 0.905-0.953), 0.814 (95% CI: 0.774-0.854) and 0.906 (95% CI: 0.879-0.933), respectively. The order efficacy of VCA-IgA in diagnosing NPC was CLIA, ELISA-2 and ELISA-1.

The AUC of EBNA1-IgA by CLIA and ELISA-2 were 0.919 (95% CI: 0.893-0.945) and 0.922 (95% CI: 0.896-0.947), respectively. The CLIA and ELISA-2 reagents of EBNA1-IgA have similar diagnostic efficacy in NPC.

### Analysis of Consistency of CLIA and ELISA in Detection of EBV Antibody

The consistency analysis of the detection results of EBV antibody kits was shown in Table 3. Generally, the negative coincidence rate among kits was higher than the positive coincidence rate. The positive and negative coincidence rates of EBNA1-IgA kits were 69.5% and 91.9%, respectively. The negative coincidence rate of VCA-IgA kits was more than 85%, but the positive coincidence rate was relatively low. The positive coincidence rates of CLIA with ELISA-1 and ELISA-2 of VCA-IgA were 50.5% and 61.0% respectively, while the positive coincidence rate of ELISA-1 and ELISA-2 of VCA-IgA was the lowest, only 49.2%.

### Sensitivity Analysis of Different Kits for Parallel Detection of Alloantibody in Diagnosis NPC

The positive coincidence rates of different EBV antibody kits were low. Further analysis of the combined detection results of EBV antibodies of different kits in NPC manifested that the kits were with a good complementary relationship. Pairwise parallel showed that the sensitivity was improved, especially the parallel detection of CLIA and ELISA-2 for VCA-IgA, CLIA and ELISA-2 for EBNA1-IgA. The sensitivity of EBV-IgA antibody in diagnosing NPC was increased to 96.5%, as shown in Table 4.

### Compliance Analysis of Results Between Different Laboratories with CLIA Kit

Laboratories at four hospitals were randomly selected, and VCA-IgA and EBNA1-IgA detection of 34 samples were repeated by CLIA. The test results were analyzed as follows: If the concentration was greater than 0.9CI, the relative deviation was calculated, and the deviation was required to be within 15%. If the concentration was less than 0.9CI, the absolute deviation was calculated, and the difference between the highest and lowest values was less than 0.25. The results showed the numbers of relative deviation and absolute deviation for VCA-IgA and EBNA1-IgA were 12 and 7, 22 and 27, respectively, and the results between hospital laboratories met the requirements, as shown in Table 5.

## Discussion

Zhongshan City is one of the high-incidence areas of NPC 13, and is also the project site of National Early Diagnosis and Early Treatment of NPC. EBV serological detection has been the most promising tool used for NPC screening. Since the 1980s, EBV antibody detection methods have successively gone through indirect immunoenzymaticassay (IEA), immunofluorescence assay (IFA) and ELISA. The first two assays have been presently rarely used, while ELISA is currently the most commonly used. However, the sensitivity and specificity of the assay need to be improved, and the detection performance of kits from different manufacturers varies 9,14,15. CLIA is a novel technique of ELISA, which replaces the traditional color substrate with chemiluminescence substrate and characterized by enhancing the luminescence signal by enzyme, and stabilizing and prolonging the luminescence signal time. It not only boosts high sensitivity of luminescent reaction, but also holds specificity of immune reaction. Previous studies have shown that CLIA embraced satisfactory sensitivity and specificity for detecting EBV IgM and IgG antibodies^16^-^18^. To further screen and optimize the detection of EBV-IgA antibody, we selected people from high-incidence areas, medium-incidence areas and low-incidence areas of NPC in China to compare the diagnostic efficacy of CLIA and ELISA for NPC, and to compare EBV antibody level in different areas.

In this study, the positive rates of EBV antibody in healthy population in different regions showed that the positive rates of CLIA, ELISA-1 and ELISA-2 reagents of VCA-IgA were quite different, with the total positive rates of 5.6%, 2.5% and 7.3%, respectively. However, the positive rates of CLIA and ELISA-2 reagents for EBNA1-IgA were similar (5.6% and 5.2%). In healthy population, the positive rate of EBV-IgA measured by CLIA was higher than ELISA. Considering that the NPC incidence rate among 20-69 year old individuals does not exceed 50 cases per 100,000 person-years even in an endemic area, a higher specificity is required for mass screening. Whether CLIA reagent for EBV-IgA is suitable for NPC screening needs further study. Previous studies have shown that ELISA for VCA-IgA has poor sensitivity in diagnosing NPC, while EBNA1-IgA has relatively high sensitivity, especially for the diagnosis of early NPC 19-21. This study also explored that in early NPC, CLIA and ELISA-2 had relatively high sensitivity to detect EBNA1-IgA, both reaching 86.4%. The sensitivity of ELISA-1 and ELISA-2 for diagnosing VCA-IgA was not satisfactory, but the sensitivity of CLIA for the detection of VCA-IgA can be further improved. The sensitivity of this kit reached 91.5% and 93.6% respectively in total NPC and advanced NPC. ROC curve analysis also showed that the diagnostic efficacy of VCA-IgA for CLIA was higher than that of the other two ELISA kits, while the diagnostic efficacy of EBNA1-IgA for CLIA was similar to that of ELISA-2 kit. A domestic ELISA compared EBV antibodies and reached a similar conclusion 22.

Consistency analysis of EBV antibody kits showed that the two kits of EBNA1-IgA had relatively excellent consistency, while the kits of VCA-IgA, especially the positive coincidence rate was low. This was consistent with Liu *et al*'s research 23. The consistency of VCA-IgA of ELISA-1 with domestic kits was low, which may be due to the different coating antigens used in different kits. VCA of EBV is a complex containing BcLF1 (P160), BFRF3 (VCA-P18), BdRF1 (VCA-p40), BLRF2 and BALF4 etc. Various VCA components contain different immune dominant domains, resulting in different levels of antibody reactions. ELISA-1 employs natural proteins purified from VCA lysated from EBV infected cells (VCA gp125), while domestic kits may use recombinant VCA protein components (VCAp18 and p23), which are less than purified virus natural proteins in the number of antigenic epitopes. However, EBNA1-IgA is merely encoded by a single gene (BKRF1), and the singleness of antigen may be one of the reasons responsible for the high consistency between kits from different manufacturers. In the future, the standards should be divided and unified calibrators should be developed according to the different peptide segments of the antigen protein to achieve an accurate consistency of the results. It is urgent to create a nation-wide golden standard serum/plasma pool in order to standardize EBV-IgA testing among laboratories and kit manufacturers 23.

The positive coincidence rates of EBV antibody detected by different kits relatively were low. Further analysis of the combined detection results of EBV antibodies of different kits for NPC manifested that the kits were with a good complementary relationship. Pairwise parallel showed that the sensitivity was improved, especially the parallel detection of VCA-IgA by CLIA and ELISA-2, EBNA1-IgA by CLIA and ELISA-2. The sensitivity of EBV-IgA antibody in diagnosis of NPC was increased to 96.5%. Whether the two different detection methods were complementary to each other, this topic will enlarge the sample size for further research. In addition, CLIA kit repeatability test found that the same specimens tested for VCA-IgA and EBNA1-IgA in four hospital laboratories, and the results showed that the differences between laboratories met the requirements.

This study also compared the positive rates of EBV antibody in healthy population in different regions. The results showed that the positive rate of EBV-IgA antibody was relatively high in high-incidence area of NPC, while there was no significant differences in the antibody positive rates between medium-incidence areas and low-incidence areas of NPC. CLIA and ELISA-2 kits for EBNA1-IgA, ELISA-1 kit for VCA-IgA all supported the above conclusions. This was consistent with the results of another large-scale population study 24, but it was different from the results of Yi Bing *et al.*^25^, considering factors such as the different sex ratio of men and women in the study population.

To sum up, CLIA method has good repeatability, higher sensitivity and similar specificity. The level of EBV-IgA antibody in healthy population in high-incidence areas of NPC is highly expressed. Detection of EBV-IgA antibody by CLIA may has good application prospect in diagnosing NPC in high-incidence areas, but further studies on improving CLIA specificity are required.

## Figures and Tables

**Table 1:** Analysis of Positive Rate of EBV Antibody Detected by CLIA and ELISA in Healthy Population

Antibody	Kit	Total population (n=1051)	high-incidence areas (n=354)	medium-incidence areas (n=318)	low-incidence areas (n=379)	Χ^2^	*P* value^#^
VCA-IgA	CLIA	59 (5.6%)	24 (6.8%)	15 (4.7%)	20 (5.3%)	1.472	0.479
	ELISA-2	77 (7.3%)	32 (9.0%)	18 (5.7%)	27 (7.1%)	2.853	0.240
	ELISA-1	27 (2.5%)	17 (4.8%)*	6 (1.9%)	4 (1.1%)	11.11	0.004
EBNA1-IgA	CLIA	59 (5.6%)	30 (8.5%)*	11 (3.5%)	18 (4.7%)	8.789	0.012
	ELISA-2	55 (5.2%)	31 (8.8%)*	11 (3.5%)	13 (3.4%)	13.37	0.001

Note: The values outside brackets were positive numbers and values in brackets were positive rates. “*” indicated high positive rate. “#” indicated the comparison of EBV antibody positive rate in high-incidence, medium-incidence and low-incidence areas of NPC.

**Table 2:** Sensitivity and Specificity of CLIA and ELISA Detection of EBV Antibody in NPC

Antibodys	Kit	Sensitivity (95%CI)			Specificity (95%CI)
		Total NPC (n=201)	Early NPC (n=44)	Advanced NPC (n=157)	
VCA-IgA	CLIA	91.5(86.6-94.8)	84.1(69.3-92.8)	93.6(88.3-96.7)	94.4(92.8-95.7)
	ELISA-2	88.6(83.1-92.5)	84.1(69.3-92.8)	89.8(83.7-93.9)	92.6(90.8-94.1)
	ELISA-1	67.7(60.7-74.0)	54.5(39.0-69.3)	73.9(66.2-80.4)	97.4(96.2-98.3)
EBNA1-IgA	CLIA	89.6(84.3-92.3)	86.4(72.0-94.3)	90.4(84.5-94.4)	94.4(92.8-95.7)
	ELISA-2	89.6(84.3-92.3)	86.4(72.0-94.3)	90.4(84.5-94.4)	94.8(93.2-96.0)

Note: Value shown as percentage.

**Table 3:** Results Consistency Analysis of EBV Antibody Detection Kit

Antibody	Kit	Positive Coincidence Rate (%)/Negative Coincidence Rate (%)	
		ELISA-1 (Reference)	ELISA-2 (Reference)
VCA-IgA	CLIA	50.5/87.2	61.0/88.6
	ELISA-1		49.2/86.3
EBNA1-IgA	CLIA		69.5/91.9

**Table 4:** Sensitivity Analysis of Different Kits for Alloantibody in Parallel Examination in Diagnosis of NPC

Different Reagent Combination		VCA-IgA (n)				EBNA1-IgA (n)
		CLIA *vs* ELISA-2	CLIA *vs* ELISA-1	ELISA-1 *vs* ELISA-2		CLIA *vs* ELISA-2
+	+	168	136	136		166
+	-	16	48	0		14
-	+	10	0	42		14
-	-	7	17	23		7
Parallel sensitivity		96.5%	91.5%	88.6%		96.5%

**Table 5:** Compliance Analysis of Results Between Different Laboratories with CLIA Kit

Number	VCA-IgA							EBNA1-IgA					
	COI-1	COI-2	COI-3	COI-4	average	relative/absolute deviation		COI-1	COI-2	COI-3	COI-4	average	relative /absolute deviation
1	2.52	2.53	2.29	2.36	2.43	4.91%*		0.13	0.16	0.35	0.11	0.19	0.24
2	3.83	3.82	3.43	3.48	3.64	5.9%*		0.16	0.2	0.21	0.18	0.19	0.05
3	0.13	0.13	0.14	0.11	0.13	0.03		0.09	0.14	0.12	0.09	0.11	0.05
4	0.26	0.28	0.26	0.28	0.27	0.02		1.01	1.09	1.05	1	1.04	3.96%*
5	0.5	0.5	0.41	0.51	0.48	0.1		1.99	2.13	2.08	2.09	2.07	2.85%*
6	1.29	1.33	1.19	1.26	1.27	4.66%*		5.17	5.5	5.3	5.2	5.29	2.82%*
7	0.21	0.22	0.22	0.2	0.21	0.02		0.14	0.22	0.3	0.12	0.2	0.18
8	0.21	0.22	0.19	0.19	0.2	0.03		0.38	0.45	0.39	0.4	0.41	0.07
9	1.65	1.59	1.37	1.55	1.54	7.83%*		0.06	0.1	0.1	0.07	0.08	0.04
10	13.1	13.6	12.7	12.6	13	3.50%*		0.15	0.21	0.2	0.13	0.17	0.08
11	1.83	1.88	1.7	1.82	1.81	4.22%*		0.1	0.17	0.21	0.12	0.15	0.11
12	0.62	0.63	0.56	0.57	0.6	0.07		0.09	0.14	0.12	0.08	0.11	0.06
13	5.17	5.06	4.24	4.86	4.83	8.59%*		27.4	28.6	26.3	26.6	27.23	3.77%*
14	9.34	9.97	8.79	9.12	9.31	5.35%*		0.06	0.07	0.18	0.07	0.1	0.12
15	15.6	15.7	14.2	14.6	15.03	4.93%		0.12	0.14	0.16	0.13	0.14	0.04
16	0.07	0.07	0.06	0.07	0.07	0.01		0.25	0.25	0.25	0.24	0.25	0.01
17	0.06	0.07	0.06	0.06	0.06	0.01		0.13	0.17	0.17	0.13	0.15	0.04
18	6.27	6.42	5.91	6.12	6.18	3.52%*		0.39	0.43	0.42	0.39	0.41	0.04
19	0.07	0.04	0.03	0.04	0.05	0.04		0.12	0.12	0.12	0.09	0.11	0.03
20	0.24	0.28	0.22	0.24	0.25	0.06		1.16	1.3	1.14	1.12	1.18	6.92%*
21	8.02	8.16	7.32	7.63	7.78	4.90%*		0.09	0.09	0.12	0.06	0.09	0.06
22	0.23	0.26	0.23	0.24	0.24	0.03		1.3	1.51	1.42	1.35	1.4	6.53%*
23	4.08	4.13	3.31	3.9	3.86	9.77%*		19.9	20.9	19.3	20.3	20.1	3.35%*
24	0.79	0.82	0.73	0.78	0.78	0.09		0.61	0.72	0.7	0.57	0.65	0.15
25	0.1	0.11	0.1	0.1	0.1	0.01		0.22	0.29	0.2	0.21	0.23	0.09
26	0.03	0.03	0.02	0.03	0.03	0.01		0.06	0.08	0.08	0.05	0.07	0.03
27	0.16	0.16	0.15	0.14	0.15	0.02		0.04	0.05	0.09	0.05	0.06	0.05
28	0.02	0.03	0.03	0.03	0.03	0.01		0.09	0.13	0.13	0.08	0.11	0.05
29	0.04	0.05	0.04	0.04	0.04	0.01		0.16	0.19	0.16	0.15	0.17	0.04
30	0.04	0.05	0.04	0.04	0.04	0.01		0.11	0.13	0.15	0.11	0.13	0.04
31	0.04	0.03	0.07	0.04	0.05	0.04		0.1	0.1	0.21	0.09	0.13	0.12
32	0.08	0.09	0.07	0.08	0.08	0.02		0.09	0.14	0.1	0.08	0.1	0.06
33	0.1	0.1	0.09	0.09	0.1	0.01		0.29	0.36	0.32	0.28	0.31	0.08
34	0.05	0.08	0.05	0.07	0.06	0.03		0.18	0.27	0.23	0.18	0.22	0.09

Note: “*”indicated relative deviation
